# Reply to Meier, R.J. Comment on “Tagliaferro et al. Introducing the Novel Mixed Gaussian-Lorentzian Lineshape in the Analysis of the Raman Signal of Biochar. *Nanomaterials* 2020, *10*, 1748”

**DOI:** 10.3390/nano13010112

**Published:** 2022-12-26

**Authors:** Alberto Tagliaferro, Elisa Padovano, Mattia Bartoli, Mauro Giorcelli

**Affiliations:** 1 Department of Applied Science and Technology, Politecnico di Torino, Corso Duca degli Abruzzi 24, 10129 Turin, Italy; 2 Faculty of Science, Ontario Tech University, 2000 Simcoe Street North, Oshawa, ON L1G 0C5 T, Canada; 3 Consorzio Interuniversitario Nazionale per la Scienza e Tecnologia dei Materiali (INSTM), Via G. Giusti, 9, 50121 Florence, Italy; 4 Center for Sustainable Future Technologies @POLITO, Istituto Italiano di Tecnologia, Via Livorno 60, 10144 Turin, Italy

## 1. Reply

First of all, we thank Dr. Meier for his thorough reading and constructive remarks [1]. His remarks helped us to both take into account some relevant references that we previously missed and get the chance to clarify some points that, despite our efforts, were not understandable enough. To make this paper more readable we organized it into paragraphs.

## 2. Background 

We agree with the remark from Dr. Meier and with the points raised in its ref. 2 [2,3]. As a matter of fact, we should have made clearer this point in the description of raw spectra analysis. A proper background drawing has always been a point of concern for us, and this remark is always part of our lectures involving Raman spectra analysis. Our approach is always (i) record the spectrum in the range 400–4000 cm^−1^; (ii) identify a single background for the whole spectrum, by a superposition of curves (including photoluminescence contribution when relevant); (iii) subtract this background from the spectrum; and (iv) focus on the region of interest. For sake of simplicity, in the paper, we limited ourselves to drawing figures of the region of interest without describing the procedure in detail.

## 3. Physical Basis 

Our use of this term has led to some confusion; hence, it has been improper. The two physically sound points are:

(1) The Debye relaxation leading to a Lorentzian lineshape does not hold from τ_0_ (attempt-to-escape frequency) to ∞ [4]. The departure from Debye relaxation becomes more relevant at short and long times, and becomes more relevant when more structural disorder is present. The presence of the disorder modifiers of the time relaxation leads to a Gaussian relaxation for fully disordered systems [4].

(2) In disordered materials, especially those with a high local coordination number (i.e., locally overconstrained), such as graphite-like carbon regions, it is common knowledge that a stretched exponential relaxation decay occurs [5,6,7]. The TCF (Time Correlation Function) of stretched relaxation has unfortunately no closed form.

## 4. Approximations 

On the basis of point 1, we assumed that the lineshape of Raman peaks in biochar can be a mixture of Gaussian and Lorentzian. We accept that no strong physical reason to impose (i) C1 constraint and (ii) symmetry of the Gaussian tail onset was identified. However, several attempts with different stretched relaxation parameters showed that the symmetrical GauLor is able to very closely mimic the lineshape arising from a stretched exponential relaxation. For this reason, we selected the symmetric Gaulor as the lineshape of interest.

## 5. Number of Components of D and G Peaks

We have carefully read the literature [8,9,10] in order to select a number of components that were at the same time meaningful and substantiated by the experiment in our case. We point out that the fit made with one D and one G component for materials such as diamond-like carbon [11] is (i) based on a minimal number of components approach, and (ii) often does not led to a nice fit of the spectrum. We picked our components in the following way:

- As far as the G contribution is concerned, we started fitting the highest temperature spectra. In such spectra a shoulder near 1610 cm^−1^ is evident, indicating the presence of a second peak beside the main one. Its attribution followed the literature [12]. The two peaks became barely distinguishable at lower temperatures due to structural disorder-related broadening, but, following its attribution, we did not see a physical reason to rule out the second peak.
- As far as the D contribution is concerned, the presence of the smaller contribution on the left tail is supported by (i) literature reports [13,14,15,16,17,18] and (ii) by the presence of slope changes in the spectrum shape that a single peak cannot justify.

As pointed out by Dr. Meier [19], with an appropriate choice of their parameters, a Lorentzian and a Gaussian can be superimposed for most of the peak range. However, this is not for the tail region as the Lorentzian has a more relevant tail. Hence, the careful background subtraction procedure that we discussed before is a key point in discriminating between the two lineshapes. 

As the pseudo-Voigt is a linear combination of Gaussian and Lorentzian over the full range, the issue raised in the previous paragraph affects the use of pseudo-Voigt too. Although a fit using pseudo-Voigt can be carried out as reported by Naylor et al. [20], the figures reported in the Supplementary Information of our paper show that there is no reasonable trend for the components across the temperature range. As a final remark, we point out that as the pseudoVoigt lineshape is a ‘computation friendly’ version of the physical meaningful Voigt lineshap, the same can be said for the Gaulor: it is a ‘computation friendly’ mimic of the lineshape determined by a stretched exponential relaxation.

## 6. Additional Remarks 

We agree that a few points remain questionable, such as the 1300 °C spectrum fit (a fit with a lower ‘left D’ component is possible). Some confusion about the labelling of D peaks might also arise due to some misprints for which we apologize.
